# Oxidative Stress and Inflammation in Sows with Excess Backfat: Up-Regulated Cytokine Expression and Elevated Oxidative Stress Biomarkers in Placenta

**DOI:** 10.3390/ani9100796

**Published:** 2019-10-14

**Authors:** Yuanfei Zhou, Tao Xu, Yinghui Wu, Hongkui Wei, Jian Peng

**Affiliations:** 1 Department of Animal Nutrition and Feed Science, College of Animal Science and Technology, Huazhong Agricultural University, Wuhan 430070, China; 2 The Cooperative Innovation Center for Sustainable Pig Production, Wuhan 430070, China

**Keywords:** sow, backfat thickness, placenta, oxidative stress, inflammation, angiogenesis

## Abstract

**Simple Summary:**

Maternal obesity is associated with increased farrowing difficulties and influences the fetus, but the effect on placental inflammation, oxidative stress, and vascular development in swine remains unclear. In this study, we tested the hypothesis that maternal obesity blocks placental vascular development associated with maternal obesity-increased placental inflammation and oxidative stress in swine. The objective of this study was to evaluate the influence of body condition of sows to placental pro-inflammatory and oxidative stress status and placental angiogenesis. we found that higher backfat thickness in sows is associated with enhanced oxidative stress, increased expression of pro-inflammation cytokines, and inhibited angiogenesis in the placenta. Therefore, reasonable control of body conditions during reproductive cycles may improve placenta development and maintain a healthy placenta environment.

**Abstract:**

In sows, excess backfat during late gestation is associated with increased farrowing difficulties and influences the fetus, but the impact of backfat thickness on placental inflammation, oxidative stress, and vascular development has not been defined. In this study, 120 sows were divided into six groups based on backfat thickness (≤16, 17–18, 19–20, 21–22, 23–24, and ≥25 mm) in late gestation. The placental lipids, reactive oxygen species (ROS), malondialdehyde (MDA), and total antioxidant capacity (TAC) levels, inflammatory-related cytokine and angiogenesis were determined. The concentrations of triglycerides, total cholesterol, low density lipoprotein cholesterol (LDL–C), and free fatty acid (FFA) linearly increased (*p* < 0.05) associated with increased late gestation backfat. ROS and MDA increased and TAC decreased (*p* < 0.05) as the backfat thickness increased. The mRNA expression of toll-like receptors (TLR) 2, TLR4, tumor necrosis factor (TNF) α, interleukin (IL)–1β, IL–6, and monocyte chemoattractant protein (MCP)–1 increased with increased backfat in late gestation. There were no differences in IL–8 and IL–10 mRNA expression among sows with different backfat thickness. Placental vessel density initially increased and then decreased with increasing backfat thickness of sows. Similarly, the mRNA levels of vascular endothelial growth factor (VEGF) were also increased and then decreased. Excessive backfat in late gestation was associated with greater oxidative stress, greater expression of proinflammatory cytokines, and decreased expression of placental angiogenic regulators.

## 1. Introduction

During gestation, the placenta plays a central role in fetal growth and development, and in the communication between dam and fetus [1]. Maternal obesity influences the placenta, and the fetus placenta is associated with reproductive disorders in human beings [2] and some animal species, like sheep [3]. Recent studies have shown that maternal obesity is associated with a lipotoxic placental environment [4] and increased inflammation [4,5] and oxidative stress [4,6] in obese women. Maternal obesity enhances the placental inflammatory signaling pathways in obese ewes at mid-gestation [7].

The placenta transports essential nutrients and substances, as well as waste products, between the maternal to fetal circulation. Placental vascular development and blood flow are critical to fetal growth and development [8]. RNA-seq analysis indicates that the genes involved in angiogenesis are significantly decreased in placenta from obese women [4]. In pregnant sheep, cotyledonary arteriole diameters are markedly greater in obese than control ewes at midgestation, and the expression of angiogenic factors is lower in obese than control ewes [3]. Therefore, placental vascular development is important to ensure optimum health of offspring.

In pigs, excess backfat during gestation is associated with an increased proportion of intrauterine growth restriction (IUGR), and decreased litter weight gain and litter size at weaning [9]. Our recent study indicated that sows with higher (≥25 mm) backfat thickness at the end of gestation demonstrate reduced litter performance, thereby indicating an effect of the lipotoxic placental environment on the number of IUGR offspring [10]. Whether pregnancy obesity has an effect on placental inflammation, oxidative stress, and vascular development in swine remains unclear. In this study, we tested the hypothesis that maternal obesity blocks placental vascular development associated with maternal obesity-increased placental inflammation and oxidative stress in swine. The objective of this study was to evaluate the influence of body condition of sows to placental pro-inflammatory and oxidative stress status and placental angiogenesis.

## 2. Materials and Methods

### 2.1. Care and Use of Animals

All animal procedures were approved by the Animal Care and Use Committee of College of Animal Science and Technology, Huazhong Agricultural University (Committee of Science and Technology). The ethical approval number is HZAUSW–2017–010. Multiparous Yorkshire sows and parity from 3–5 were studied. One hundred and twenty sows were divided into six groups on the basis of backfat thickness (≤16, 17–18, 19–20, 21–22, 23–24, and ≥25 mm) at 109 day of gestation. Backfat thickness was measured at the last rib (P2; 6.5 cm from the midline over the last rib) [11] using ultrasound (PIGLOG105, SFAK-Technology, A Mode Scanner, SFK Technology A/S, Helver, Denmark) [10]. During gestation, sows were housed individually in gestation stalls. Sows were moved from the gestation stalls to the farrowing rooms on day 109 of gestation and then housed individually in farrowing crates with stalls in pens, and the backfat thickness of sows was recorded. Each crate was equipped with electronic feeders. The farrowing room temperature was maintained at approximately 18–20 °C with a water-cooling ventilation system.

### 2.2. Tissue Collection

Wilson’s method was used to ensure that the collected placentas belonged to their respective newborn piglets [12]. When the sow farrowed, each umbilical cord was tied with a long silk line which was attached to a numbered tag to match the birth order of the piglets [13]. Six to eight placental tissues were collected per sow. A total of 67 sows’ placenta samples were collected. Placenta sample was obtained from placentomes of similar size within 5–10 cm of umbilical site, and five to six samples were collected from each placenta. Then, they were immediately frozen in liquid nitrogen and stored at −80 °C until used for analysis.

### 2.3. Lipid Analyses

Lipids were extracted from 200–400 mg of placental tissue with chloroform–methanol (2:1, vol/vol) [14]. The concentrations of the triglyceride, total cholesterol, and the low-density lipoprotein cholesterol (LDL–C) in the placenta were measured by enzyme assay. The protein content was measured using a bicinchoninic acid (BCA) protein assay kit (BCA Protein Assay Kit, Nanjing Jiancheng Bioengineering Institute; Nanjing, China).

### 2.4. Free Fatty Acid (FAA) Assay

Placenta total FFA content was analyzed using the colorimetric method [14,15]. The FFA content was assessed by a fluorometric kit (Nanjing Jiancheng Bioengineering Research Institute, A042, Nanjing, China) in accordance with the instructions of manufacturer.

### 2.5. Reactive Oxygen Species (ROS) and Lipid Oxidation Assay

Placental tissue (200 mg) was homogenized. The reactive oxygen species (ROS) concentration analysis was performed by the chemiluminescence (CL) assay using luminol as an indicator of radical formation. The tissue samples were recorded at room temperature using an LB 940 luminometer (Berthold Technologies, Bad Wildbad, Germany) in the presence of enhancers as described previously [16]. The contents of malondialdehyde (MDA) were assayed using colorimetric methods with a spectrophotometer (Biomate 5, Thermo Electron Corporation, Rochester, NY, U.S.A.). Total antioxidant capacity (TAC) was measured with a commercial kit (Nanjing JianCheng Bioengineering Institute, A015, Nanjing, China) as per manufacturer’s instructions.

### 2.6. Placenta Histology and Vascular Density Determination

Fresh placental tissues were collected and fixed in 4% neutral buffered formalin solution (HT501; Sigma, St. Louis, Missouri, USA). The tissues were sliced into 5 μm-thick sections and stained with hematoxylin and eosin. For each of the 5-μm sections, the total number of vessels in the placental stroma areas were determined, then corrected with the total placental stroma areas measured (per unit area as mm^2^). The number of vessels could be determined via image analysis, and values were then averaged across 3 slices of one placentome were then averaged [17]. Five placentomes were selected conceptuses from litters in each selection group on parturition day.

### 2.7. Real-Time PCR

Total RNA was extracted from 200 mg of homogenized placental tissue using TRIzol reagent (Invitrogen^TM^, Carlsbad, California, USA) according to manufacturer’s instructions and followed by DNase digestion using a DNA-free kit (Applied Biosystems, Thermo Fisher Scientific, California, USA) according to the manufacturer’s instructions. Total RNA was purified using RNeasy MinElute Spin Columns (Qiagen). Purity level and concentration of isolated total RNA was measured using NanoDrop^®^ ND-1000 UV-Vis spectrophotometer (Thermo Fisher Scientific Inc., Waltham, Massachusetts, USA), and then transcribed into cDNA by the first strand cDNA synthesis kit (TOYOBO, Osaka, Japan). Relative mRNA levels of genes were quantified by using a Bio-Rad CFX Connect™ Real-Time PCR Detection System (Bio-Rad, Richmond, CA, USA). β–actin mRNA levels were similarly measured and served as the reference gene. Real-time PCR was performed according to our previous study [18] and the mean of the triplicate cycle thresholds (CT) of the target gene was normalized to the mean of triplicate CT of the reference β–actin using the calculation formula “2^CT^_β-actin_^−CT^_target gene_,” indicating a relative value as a fraction of the target gene. The sequences of the primers are listed in Supplementary Table S1.

### 2.8. Statistical Analysis

Statistical analyses were conducted using the PROC MIXED procedure of SAS 9.2 (SAS Inst. Inc., Cary, NC, USA). Backfat thickness at 109 day of gestation, parity, and the interaction between parity and the categorized backfat thickness at 109 day of gestation were specified as fixed effects. Sow and boar were random effects. The response variables were related measurements, including triglyceride, cholesterol, LDL–C, and FFA. Regression analyses were performed to evaluate the linear and quadratic effects of backfat thickness on day 109 of gestation. Placental ROS, MDA, and TAC levels, gene expression, and vessel density data were analyzed the using one-way ANOVA with Duncan’s multiple comparison test. For this model, we designated placental sample from each individual dam as the repeated measures factor. Graphing was performed with the use of GraphPad Prism version 6 (GraphPad Software, San Diego, CA, USA). *p* < 0.05 was considered statistically significant.

## 3. Results

### 3.1. Placental Lipids in Different Backfat Thickness Sows

The placental lipid results are shown in Table 1. Triglyceride, cholesterol, and low-density lipoprotein cholesterol (LDL–C) concentrations and free fatty acid content exhibited a linear increase (*p* < 0.05) as the backfat thickness at late of gestation increased.

### 3.2. Placental Reactive Oxygen Species (ROS), Malondialdehyde (MDA) and Total Antioxidant Capacity (TAC) Levels in Different Backfat Thickness Sows

**Figure 1:**
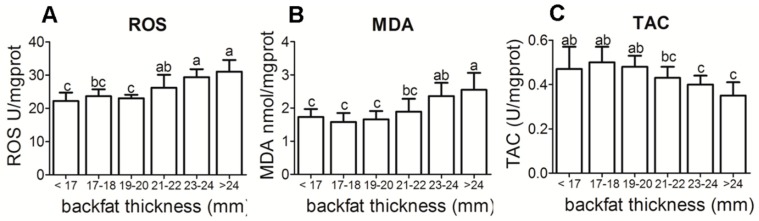
Antioxidant status in placenta of different backfat thickness sows. (**A**) Placental reactive ROS level in different backfat thickness sows; (**B**) Placental reactive MDA level in different backfat thickness sows; (**C**) Placental reactive TAC level in different backfat thickness sows. ROS—reactive oxygen species; MDA—malondialdehyde; TAC—total antioxidant capacity. Each group had 10–12 sows, 4–5 placenta samples were analyzed per sow, mean ± SEM. Different letter showed significant difference (*p* < 0.05).

ROS, MDA levels, and TAC at different backfat thickness were observed in the placenta of sows (Figure 1A–C). A significant increase in the levels of placental ROS (*p* < 0.05) and MDA (*p* < 0.05) were associated with increased backfat thickness. TAC values decreased as BF thickness increased (*p* < 0.05) as the backfat thickness increased.

### 3.3. Placental Inflammatory-Related Cytokine Gene Expression in Different Backfat Thickness Sows

**Figure 2:**
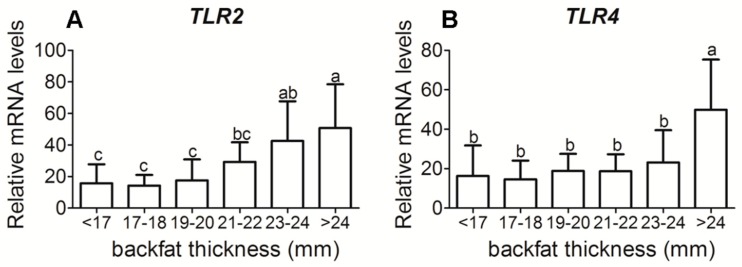
Toll-like receptor 2 and 4 mRNA expressions in placenta tissue of different backfat thickness sows. (**A**) Placental reactive Toll-like receptor (TLR) 2 mRNA level in different backfat thickness sows; (**B**) Placental reactive *TLR4* mRNA level in different backfat thickness sows. Each group had 10–12 sows, 4–5 placenta samples were analyzed per sow, mean ± SEM. Different letter showed significant difference (*p* < 0.05).

**Figure 3:**
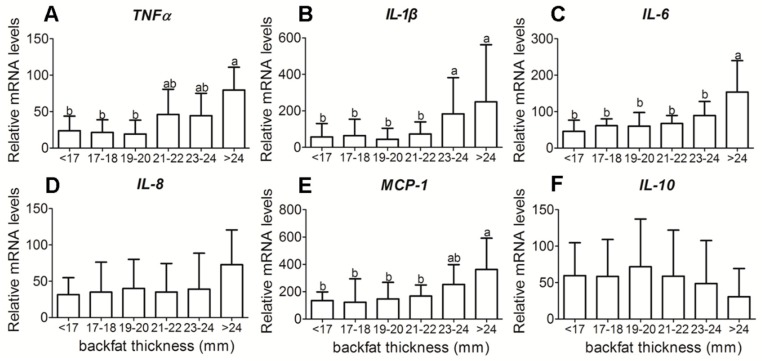
Pro-inflammatory cytokines and anti-inflammatory cytokines mRNA expressions in placenta tissue of different backfat thickness sows. (**A**) Placental reactive tumor necrosis factor α (*TNF*α) mRNA level; (**B**) Placental reactive interleukin (IL)-*1β* mRNA level; (**C**) Placental reactive IL-6 mRNA level; (**D**) Placental reactive IL-8 mRNA level; (**E**) Placental reactive monocyte chemoattractant protein-1 (MCP-1)mRNA level; (**F**) Placental reactive *IL-10* mRNA level mRNA level;. Each group had 10–12 sows, 4–5 placenta samples were analyzed per sow, mean ± SEM. Different letter showed significant difference (*p* < 0.05).

Sows with a greater backfat had increased placenta mRNA levels of both toll-like receptors (*TLR*) *2* (*p* < 0.05) and *TLR4* (*p* < 0.05), key pathogen recognition receptors (Figure 2). With increased backfat thickness, mRNA expression of pro-inflammatory cytokines, TNFα, interleukin (IL)–1β, IL–6, and monocyte chemoattractant protein (MCP)–1 exhibited an increase (Figure 3A–C,E), while IL–8 was not significantly different (Figure 3D). The mRNA expressions of anti-inflammatory cytokine IL–10 were not significantly different either (Figure 3F).

### 3.4. Placental Vessel Density and Angiogenic Regulators Gene Expression in Different Backfat Thickness Sows

**Figure 4:**
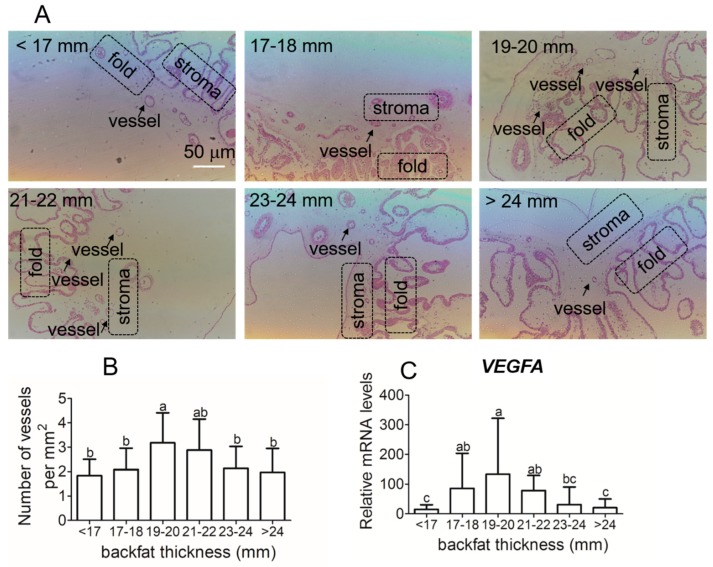
Average numbers of capillary per mm^2^ placenta tissues and vascular endothelial growth factor (VEGF)A mRNA expressions in placenta tissue of different backfat thickness sows. (**A**) placenta tissue slices. (**B**) to quantify villous capillary density. (**C**) VEGFA mRNA expressions. Each group had 10–12 sows, and 4–5 placenta samples were analyzed per sow, mean ± SEM. Different letter showed significant difference (*p* < 0.05). The arrows indicate the placental vessels in stroma (200× magnification).

Representative images of the placental vessels are presented in Figure 4A. Placental vessel density in sows with backfat thickness of 19–22 mm was significantly higher than sows with backfat less than 19 mm or with backfat greater than 23 mm (Figure 4B). The mRNA expression of angiogenesis related factors, vascular endothelial growth factor (VEGF), was similar to vessel density (Figure 4C).

## 4. Discussion

During the first two-thirds of gestation, adipose fatty acid synthesis increases and lipid accumulates [19]. During the last third of gestation, the increase in maternal fat depot accumulation stops, and adipose tissue fatty acid synthesis and lipoprotein lipase (LPL) activity decrease [19]. Previous studies have shown that maternal obesity results in reduced uptake and storage of fatty acids, along with increases in lipolysis and increases of circulating non-esterified fatty acid (NEFA) [20,21]. Thus, the excessively high-circulating NEFA concentrations lead to insulin resistance and promote ectopic fat accumulation in extra-adipose tissues such as liver, muscle, heart [22], and placenta [4]. Triglyceride, cholesterol, LDL–C, and free fatty acid concentrations were elevated along with increasing backfat thickness of sows in late gestation. Zhu et al. (2010) reported that maternal obesity elevated the levels of maternal plasma and fetal plasma cholesterol and triglycerides in the ewes [7]. In pigs, the obese Iberian sows were fatter and had greater plasma concentrations of leptin, triglycerides, and cholesterol than their lean crossbred sows [23]. Lipid storage in non-adipose tissue, such as liver, muscle, and placenta is associated with the occurrence of metabolic disorders. The excessive oxidation of fatty acids leads to ROS production. Our recent study indicated that sows with greater backfat thickness (≥21 mm) in late gestation induces lipotoxic placental environment [10], which is associated with declining reproductive performance [10]. It is further indicated that in maternity obesity, the higher m^6^A modification displayed in the genes related to placental development, lipid metabolism, and angiogenesis [13]. The related genes expression was reduced, which could be associated with m^6^A demethylase fat mass and obesity-associated (FTO) [13].

Maternal obesity is associated with oxidative stress and high levels of ROS [6]. The high lipid levels and oxidative stress lead to the production of superoxide ions, hydroxyl radicals, and hydrogen peroxide [24]. Our data indicated that ROS levels and MDA, a lipid peroxidation marker, were increased in the placenta of sows with increased backfat thickness. Lipid oxidation can influence placental development, lipid metabolism, and transport, and can also affect fetal developmental pathways [4]. In addition, our findings suggest that antioxidant capacity was reduced in placenta from higher backfat thickness sows. The altered antioxidant response can lead to increased lipid peroxidation [25]. In obese women, TAC was decreased by 25%, indicating an impaired capacity to deal with oxidative stress in placenta [4]. In contrast, there have been other reports that placental antioxidant capacity was increased in parallel with increased oxidative stress in Type 1 diabetic pregnancies. One possible reason for this is that the placenta can adapt to higher levels of oxidative stress [26]. Hence, our data show that a greater backfat thickness of sows is associated with increased lipids concentrations and oxidative stress and reduced total antioxidant capacity in the placenta.

Maternal obesity has been associated with low-grade metabolic inflammation. Some studies have shown that maternal obesity is associated with elevated proinflammatory cytokines during pregnancy and in the placenta [4,7,27,28]. In sheep, maternal obesity increased mRNA levels of TLR2 and 4, and pro-inflammatory cytokines TNFα, IL–1β, IL–6, and IL–8 in placenta [7]. Meanwhile, inflammatory signaling pathways, c–Jun N–terminal kinase (JNK)/c–Jun and nuclear factor kappa–light–chain–enhancer of activated B cells (NF–κB) were up-regulated in the placenta of obese sheep [7]. In women, maternal obesity may increase expression of inflammatory cytokines IL–1, IL–6, and TNF–a, and have an impact on maternal and fetal health [5,29]. In the present study, we measured the expression profile of pro-inflammatory and anti-inflammatory genes directly from the placenta of sows. We found a significant increase in the mRNA expression of TNFα, IL–1β, IL–6, and MCP–1. However, we found no increase in the mRNA expression of IL–8. These results suggested that maternal obesity is associated with placental inflammation.

In the present study, we evaluated the placenta tissue of biomarkers of inflammation and oxidative stress of different body conditions of sows. Placental oxidative stress and inflammation is clearly associated with multiple adverse pregnancy outcomes, including miscarriage, recurrent pregnancy loss, embryopathy, pre-eclampsia, fetal growth restriction, preterm premature rupture of membranes, and gestational diabetes [30]. Notably, oxidative stress and inflammation markers are seen in pregnancies complicated for altered reproduction and development.

The placenta is the organ that transports nutrients, respiratory gases, and waste between the maternal and fetal circulation throughout the placental vascular network. Previous studies have shown that obesity during pregnancy inhibits angiogenesis [3,4]. In obese women, hypoxia-inducible factor (HIF)–1α and VEGF–A levels were significantly decreased in placenta compared to lean women [4]. Similarly, our data indicate that the reduced angiogenesis and VEGF expression only occurs when compared to sows with moderate backfat thickness. Vessel density in placentas from sows with backfat less than 19 mm does not differ from those with backfat greater than 23 mm. Similarly, low VEGF expression is observed in sows with backfat thickness less than 17 mm. Care needs to be taken to indicate that this reduction in angiogenesis in high backfat thickness sows is relative to those with moderate backfat thickness.

## 5. Conclusions

In conclusion, we report a novel finding that higher backfat thickness in sows is associated with enhanced oxidative stress, increased expression of pro-inflammation cytokines, and inhibited angiogenesis in the placenta. Therefore, reasonable control of body conditions during reproductive cycles may improve placenta development and maintain a healthy placenta environment.

## Figures and Tables

**Table 1:** Triglyceride and cholesterol and free fatty acid concentrations in placenta.

Item	Backfat Thickness (mm) of Sows at 109 Day of Gestation						SEM	*p*-Value	
	≤16	17–18	19–20	21–22	23–24	≥25		L ^3^	Q ^4^
Number, *n*	10	12	12	11	12	10			
Triglyceride, mmol/gprot	0.17	0.16	0.17	0.18	0.22	0.25	0.01	0.02	0.15
Cholesterol, mmol/gprot	0.11	0.10	0.10	0.13	0.15	0.19	0.01	0.02	0.32
LDL-C ^1^, μmol/gprot	25.65	27.42	27.53	32.84	38.03	42.45	0.52	0.01	0.52
FFA ^2^, ng/mgprot	96.18	94.94	96.07	99.32	105.66	119.24	1.26	0.03	0.37

^1^ LDL–C—low-density lipoprotein cholesterol; ^2^ FFA—free fatty acid; ^3^ L—Linear discriminant analysis; ^4^ Q—Quadratic discriminant analysis.

## Supplementary Materials

The following are available online at https://www.mdpi.com/2076-2615/9/10/796/s1, Table S1: Primer sets used for real-time quantitative PCR.
