# Supplementary material for: Oxidative Stress and Inflammation in Sows with Excess Backfat: Up-Regulated Cytokine Expression and Elevated Oxidative Stress Biomarkers in Placenta
Source: Animals (Basel). 2019 Oct 14;9(10):796. doi: 10.3390/ani9100796 (PMC6826773; doi:10.3390/ani9100796)
Supplement: Supplementary file 1 [file animals-09-00796-s001.pdf]

Supplementary data

Table S1. Primer sets used for real-time quantitative PCR.

Gene Name	Accession Number	Sequence (sense/antisense 5′–3′)	Product Size
IL1β	NW_018085011.1	AAAGGGGACTTGAAGAGAG<br>CTGCTTGAGAGGTGCTGATGT	286 bp
IL6	NM_214399.1	AAGGTGATGCCACCTCAGAC<br>TCTGCCAGTACCTCCTTGCT	151 bp
IL8	M86923.1	TTGCCAGAGAAATCACAGGA<br>TGCATGGGACACTGGAAATA	341 bp
IL10	NM_214041.1	CTGCCTCCCACTTTCTCTTG<br>TCAAAGGGGCTCCCTAGTTT	200 bp
MCP1	NC_010454.4	GTCCTTGCCCAGCCAGATG<br>CGATGGTCTTGAAGATCACTGCT	148 bp
TNFα	NM_213969.1	CACCACGCTCTTCTGCCTACTG<br>TTGAGACGATGATCTGAGTCCTTGG	115 bp
TLR2	NM_213761.1	TGCTTTCCGAGAACTTTGT<br>GCAGAATGAGGATGGCG	129 bp
TLR4	NM_001113039.2	AAGGTTATTGTCGTGGTGT<br>CTGCTGAGAAGGCGATAC	161 bp
VEGFA	NM_214084.1	ATGGCAGAAGGAGACCAGAA<br>ATGGCGATGTTGAACTCCTC	224 bp
β-actin	DQ452569.1	CCAGGTCATCACCATCGG<br>CCGTGTTGGCGTAGAGGT	158 bp
